# High-Density Lipoprotein (HDL) Triglyceride and Oxidized HDL: New Lipid Biomarkers of Lipoprotein-Related Atherosclerotic Cardiovascular Disease

**DOI:** 10.3390/antiox9050362

**Published:** 2020-04-26

**Authors:** Fumiaki Ito, Tomoyuki Ito

**Affiliations:** 1 The Institute of Prophylactic Pharmacology, Shinagawa, Tokyo 140-0001, Japan; 2 Physical Medicine and Rehabilitation, Tanabe Memorial Hospital, Kyotanabe-City, Kyoto 610-0331, Japan; rinito@par.odn.ne.jp; 3 Department of Rehabilitation Medicine, Kyoto Prefectural University of Medicine, Kyoto 602-8566, Japan

**Keywords:** cardiovascular disease, atherosclerosis, high-density lipoproteins, low-density lipoproteins, reactive oxygen species and lipid peroxidation, triglycerides, chronic inflammation

## Abstract

Lipid markers are well-established predictors of vascular disease. The most frequently measured lipid markers are total cholesterol, high-density lipoprotein (HDL)-cholesterol (HDL-C), LDL cholesterol (LDL-C), and triglyceride. HDL reduces atherosclerosis by multiple mechanisms, leading to a reduced risk of cardiovascular disease, and HDL-C, as a metric of HDL quantity, is inversely associated with cardiovascular disease, independent of LDL-C. However, the quality of the HDL appears to be more important than its quantity, because HDL loses its antiatherogenic functions due to changes in its composition and becomes “dysfunctional HDL”. Although there is evidence of the existence of “dysfunctional HDL”, biomarkers for monitoring dysfunctional HDL in clinical practice have not yet been established. In this review, we propose a new lipid panel for the assessment of dysfunctional HDL and lipoprotein-related atherosclerotic cardiovascular disease. The lipid panel includes the measurement of lipid peroxide and triglyceride contents within HDL particles.

## 1. Introduction

Multiple lines of evidence have established that LDL cholesterol (LDL-C) and other apolipoprotein B (apoB)-containing lipoproteins are directly implicated in the development of atherosclerotic cardiovascular disease [1,2]. Therefore, LDL-C levels are associated with the rate at which cardiovascular events occur. On the other hand, high-density lipoprotein (HDL)-cholesterol (HDL-C) is inversely associated with the risk of coronary heart disease and is a key component of predicting cardiovascular risk [3,4]. However, HDL-C-elevating drugs such as niacin, fibrates, and cholesteryl ester transfer protein (CETP) inhibitors have failed to decrease cardiovascular risk when tested in patients on statin therapy [5]. It was also reported that the antiatherogenic effects of HDL are impaired in patients with diabetes, coronary heart disease or chronic kidney dysfunction compared with those of HDL from healthy subjects [6,7]. Therefore, the protective effects of HDL against cardiovascular risk cannot be fully explained by the HDL-C concentration. Because HDL has many biological functions that may contribute directly or indirectly to the prevention of cardiovascular disease, the functional quality of HDL is a better determinant of HDL cardiovascular protection than the concentration of HDL in the peripheral circulation [8]. 

HDLs are a highly heterogeneous lipoprotein family, consisting of several subclasses differing in size, shape, and lipid and protein composition. The particle number and size distribution of HDLs and their lipid and protein composition can be characterized by nuclear magnetic resonance (NMR) and mass spectrometry spectroscopy. Several large-scale clinical trials indicated that a reduced concentration of circulating HDL particles can be superior to HDL-C concentration as a predictor of cardiovascular disease [9]. Furthermore, metrics of HDL functionality, such as HDL cholesterol efflux capacity, may represent an obvious alternative to HDL-C concentration in the peripheral circulation, although the different cellular functions of HDL are weakly correlated with each other and are determined by different structural components [10]. However, NMR analysis and cell-based assay of HDL functionality have disadvantages in terms of the complexity of the methodologies and their time-consuming nature. This article focuses on simpler and clinically applicable assays for the assessment of HDL functionality. 

## 2. Dysfunctional HDL and Oxidative Stress

### 2.1. Dysfunctional HDL 

HDL and/or its most abundant protein constituent, apolipoprotein A-I (apoA-I), have antiatherogenic functions. The loss of this antiatherogenic function of HDL, often called dysfunctional HDL, occurs due to changes in the amount and type of proteins and lipids bound to the HDL particle. For example, the functional loss of HDL can be attributed to its compositional change, as evidenced by the reduced content of sphingosine-1-phosphate in HDL isolated from patients with coronary artery disease [11]. Furthermore, a recent study has suggested that HDL-associated enzymes, paraoxonase 1 and myeloperoxidase (MPO), are potential indicators of dysfunctional HDL and risk the stratification of coronary heart disease [12,13]. 

**Figure 1:**
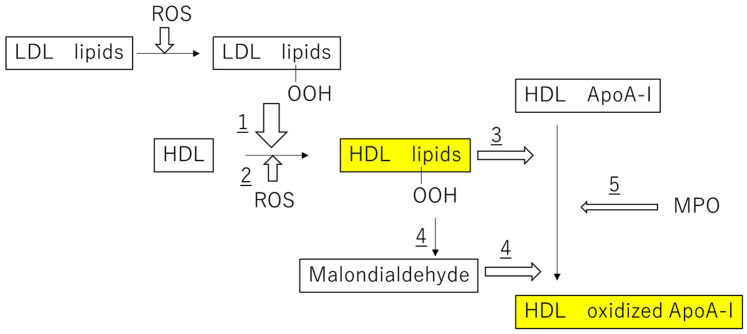
Increased oxidation of lipid components and ApoA-I in high-density lipoprotein (HDL) particles.

The oxidative modification of lipid and protein constituents in HDL particles is another cause of the functional loss of HDL, because these HDL constituents are known to be susceptible to oxidative modifications by a variety of oxidants, such as peroxyl and hydroxyl radicals, aldehydes, and various MPO-generated oxidants [14]. Thus, as summarized in Figure 1, HDL is considered to lose its antiatherogenic functions by multiple oxidative reactions.

### 2.2. Antiatherogenic Functions of HDL

Oxidative stress induced by the generation of excess reactive oxygen species (ROS) in the vascular wall has emerged as a critical, final common mechanism in atherosclerosis. Major ROS-producing systems include nicotinamide adenine dinucleotide phosphate (NADPH) oxidase, xanthine oxidase, the mitochondrial electron transport chain, MPO, and uncoupled endothelial nitric oxide (NO) synthase [15]. Very early in the atherosclerotic process, a disturbed blood flow induces an increase in endothelial permeability to LDL and ROS production in the endothelial cells. These increased levels of ROS promote LDL oxidation, resulting in a high level of oxidized LDL (ox-LDL), leading to the apoptosis of vascular endothelial cells and subsequent endothelial dysfunction. Ox-LDL can also promote oxidation in the vascular wall, resulting in a high level of lipid hydroperoxides (LOOH) in LDL (Figure 1). Subsequently, blood monocytes are recruited into the intima, where they differentiate into macrophages and take up LDL, very low-density lipoprotein (VLDL), and oxidized lipoproteins by the scavenger-receptors [16]. When macrophages take up excess lipids, they differentiate into lipid-laden foam cells. The representative antiatherogenic functions associated with HDL are its antioxidative activity against ox-LDL and its promotion of reverse cholesterol transport from lipid-laden foam cells. 

### 2.3. Oxidation of Lipids and apoA-I in HDL Particles 

LOOH in ox-LDL is transferred from LDL to HDL either spontaneously or mediated by lipid transfer proteins, including CETP (reaction 1 in Figure 1). Thus, HDL potently inhibits the accumulation of LOOH in LDL and mediates protection from oxidative damage induced by ROS, as LOOH in HDL is increased due to the transfer reaction. In addition, the LOOH content in HDL increases through the direct oxidation by ROS (reaction 2 in Figure 1). The direct oxidation of HDL lipids actually occurs in vivo, because the lipids of HDL are initially oxidized in preference to those in LDL when human plasma is exposed to aqueous peroxyl radicals [17]. As a consequence of reactions 1 and 2, HDL particles constitute a major transport vehicle of oxidized lipids in human plasma, although LOOHs and their corresponding hydroperoxides can be rapidly removed in plasma via scavenger receptor class B-I-mediated selective uptake by the liver [18].

HDL is highly heterogeneous and carries a large number of different proteins. ApoA-I, the most abundant protein component in HDL, contributes to the inhibition of LOOH accumulation in both LDL and HDL. The key role of apoA-I in the antioxidative activity of HDL is supported by the observation that reconstituted HDL, containing only purified apoA-I and phospholipid, but devoid of other protein components, exerts a similar antioxidative action compared to small dense HDL subfractions [19]. Phospholipid-OOH (PLOOH) in LDL is initially transferred from LDL to HDL, and this PLOOH is subsequently reduced by the redox-active methionine (Met) of apoA-I with the formation of PLOH and methionine sulfoxide MetS(O) (reaction 3) [20]. PLOOH is preferentially processed by HDL compared to cholesterol ester-OOH, because PLOOH is located in the surface monolayer of LDL and thus is more readily accessible for transfer to HDL [20]. 

Various bioactive aldehydes, such as malondialdehyde (MDA), 4-hydroxynonenal (4-HNE), and acrolein, are generated by free radical-mediated lipid peroxidation [21,22,23]. HNE forms adducts with three different amino acyl side chains, namely Cys, His, and Lys residues, via Michael addition either to thiol (−SH) or to amino (−NH_2_) groups [24], whereas MDA reacts in vivo with protein-bound Lys residues to form dihydropyridine (DHP)-type adducts [25,26]. Several studies have reported that the modification of HDL with these reactive aldehydes impairs HDL’s antiatherogenic functions. For example, the conversion of Lys-226 to N^ϵ^-(3-methylpyridinium) lysine by acrolein is associated quantitatively with decreased cholesterol efflux from cells via the ATP-binding cassette transporter A1 pathway [27,28]. Furthermore, acrolein-modified HDL promotes the higher accumulation of neutral lipids in murine macrophages, as judged by Oil Red O staining compared to native HDL, suggesting that acrolein modification of HDL produces a dysfunctional particle that may ultimately promote atherogenesis by impairing the cholesterol transport function of HDL [28]. Therefore, LOOH in HDL could impair a variety of HDL’s antiatherogenic functions by oxidizing apoA-I via a reactive aldehyde-mediated reaction (reaction 4). 

Not only activated macrophages, but also T lymphocytes, platelets, and neutrophils contribute to atherosclerosis. MPO is released into the extracellular space due to neutrophil activation, and MPO-derived oxidants can cause the MPO-dependent oxidization of LDL and HDL [29]. MPO catalyzes the chlorination and nitration of apoA-I at its Lys, Met, and Trp residues in human atherosclerotic lesions and affects apoA-I-mediated HDL functions (reaction 5). For example, site-specific modification of the Trp residues of apoA-I generates a dysfunctional apoA-I with impaired cholesterol efflux acceptor activity and pro-inflammatory function [30,31]. Because the association of elevated oxidized Trp_72_-ApoA-I levels with increased cardiovascular disease risk has been reported [32], circulating oxidized Trp_72_-apoA-I levels may serve to predict cardiovascular disease risk.

### 2.4. Association of HDL–LOOH with Arterial Stiffness and Chronic Inflammation

**Figure 2:**
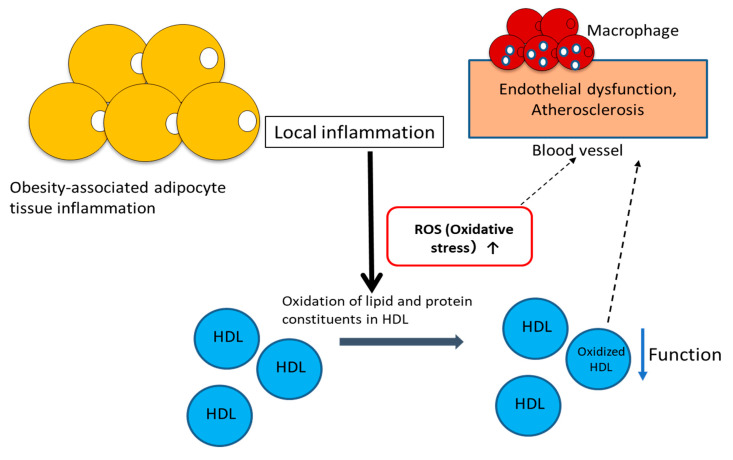
Reactive oxygen species (ROS) and ROS-induced oxidative modification of HDL cause endothelial dysfunction and progression of atherosclerosis.

Because HDL–LOOH is considered a biomarker of HDL dysfunction and cardiovascular risk, many assay methods to assess lipid peroxidation have attracted great interest. Recently, we developed a new method (termed the “Fe-ROMs test”) for the assessment of the LOOH content in blood and found that most of the LOOH measured by this test is included in HDL [33]. Thus, the Fe-ROMs test provides a simple method for measuring plasma HDL–LOOH levels. We used this test to examine the association of HDL–LOOH levels with vascular injury in diabetic patients [29]. In that study, the cardio–ankle vascular index (CAVI) was used as an index of arterial stiffness, because CAVI and brachial–ankle pulse wave velocity are widely used clinically for the early evaluation of functional and structural changes in the vascular wall [34,35]. The results showed that the ratio of HDL–LOOH to HDL-C, but not the HDL–LOOH content, correlated with CAVI. Because HDL–LOOH content would be expected to be dependent on the HDL concentration, this ratio is considered to be a better index of vascular dysfunction than just the HDL–LOOH content. Our group also found the association of this ratio, but not HDL–LOOH content, with the visceral fat area (VFA), which is a good surrogate marker of obesity-related disorders, and high-sensitivity C-reactive protein (hs-CRP), a marker of inflammation, by performing a Pearson correlation analysis on data collected from patients with type 2 diabetes mellitus (T2DM) [29]. Therefore, as summarized in Figure 2, the HDL–LOOH/HDL-C ratio seems to be a predictor of chronic low-grade inflammation associated with increased adipose tissue mass and local vascular changes. Low-grade local inflammation induces ROS generation and the synthesis and secretion of pro-inflammatory cytokines, and subsequently increases the ROS-mediated lipid peroxidation of HDL. Consequently, it is likely that increased HDL–LOOH levels, together with oxidative stress, cause vascular injury. Although the measurement of oxidized HDL, i.e., LOOH in HDL, is recommended as the predictor of dysfunctional HDL, the LOOH/HDL-C ratio may be a more reliable biomarker for monitoring dysfunctional HDL in both the clinical setting and experimental studies. 

## 3. Dysfunctional HDL and Triglyceride Level 

Numerous studies have demonstrated that elevated triglyceride (TG) levels are associated with an increased risk of coronary heart disease [36,37]. Hypertriglyceridemia, namely elevated levels of TG-rich lipoproteins and their remnants, results from either increased production or decreased catabolism of TG-rich lipoproteins. CETP promotes the transfer of CE from CE-rich lipoproteins, i.e., LDL and HDL, towards TG-rich lipoproteins, mainly VLDL, coupled to a net flux of TG from VLDL to CE-rich lipoproteins [38,39]. Therefore, in the presence of elevated TG levels, CETP increases the TG/CE ratio of CE-rich lipoproteins i.e., HDL, and lowers this ratio in TG-rich lipoproteins. TG-enriched HDL produces a higher number of smaller HDL particles with higher catabolic rates and, as a result, lowers the levels of HDL particles [5,39]. Moreover, the elevated transfer rate of CE from HDL to VLDL particles increases the number of large VLDL particles, which are typically catabolized to the more atherogenic small-dense LDL particles [40,41]. Patients with diabetes, obesity or metabolic syndrome due to insulin resistance tend to have low HDL-C and a high number of small and dense LDL particles because of their lower lipoprotein lipase activity and TG enrichment [5]. 

As described above, hypertriglyceridemia causes higher levels of small, dense LDL and lower levels of HDL. In addition, it increases the TG-enriched HDL, but the effect of TG enrichment on HDL functions were not intensively studied until recently. A recent nested case-control study reported that TG within HDL particles is positively associated with myocardial infarction (MI) and ischemic stroke (IS), whereas cholesterol in HDL is inversely associated with the risk of MI and IS [42]. On the other hand, both TG and cholesterol within apolipoprotein B-containing lipoproteins were shown to be positively associated with MI and IS. Therefore, the positive association of TG in HDL lipoproteins with MI and IS differed from the inverse association of cholesterol in HDL lipoproteins, indicating that TG and cholesterol in HDL particles may have opposing relationships with respect to the risk of MI [42]. Furthermore, another group observed a strong association between HDL–TG and metabolic disturbances such as diabetes, metabolic syndrome, and obesity [5]. Thus, the measurement of HDL–TG provides additional information about vascular risk. 

**Figure 3:**
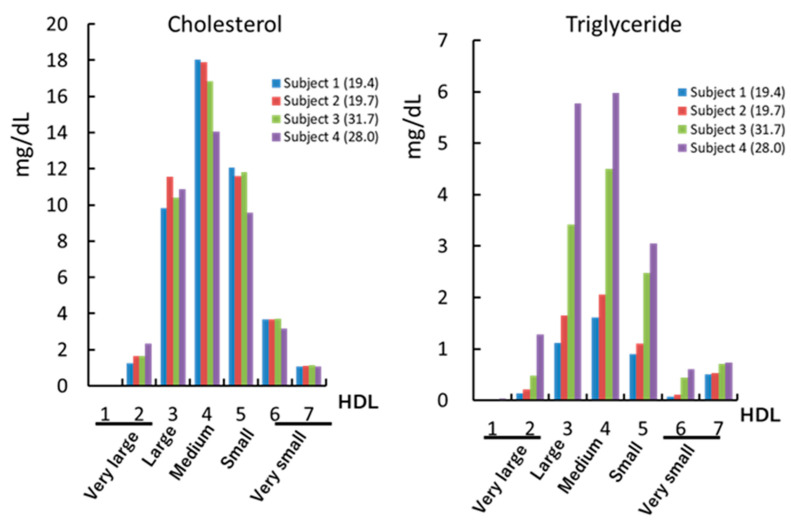
Concentrations of cholesterol (left panel) and triglyceride (right panel) in HDL subclasses. HDL isolated from four subjects were analyzed by using gel-permeation high-performance liquid chromatography. LOOH-HDL levels in subjects 1, 2, 3, and 4 are 19.4 mOD/min, 19.7 mOD/min, 31.7 mOD/min, and 28.0 mOD/min, respectively.

We previously measured HDL–LOOH levels in four healthy subjects by using the Fe-ROMs test. Two of them had higher levels of HDL–LOOH, and the other two had lower ones. We determined the concentrations of HDL-C and HDL–TG in four subjects and detected similar concentrations of HDL-C in all of them (45.99, 47.50, 45.61, and 41.11 mg/dL in subjects 1, 2, 3, and 4, respectively) [33]. However, concentrations of HDL–TG were different among the subjects: 4.35 and 5.67 mg/dL in two subjects with lower HDL–LOOH levels and 12.05 and 17.44 mg/dL in the other two subjects with higher HDL–LOOH levels. A more detailed analysis, in which HDLs were separated by gel-permeation high-performance liquid chromatography (GP-HPLC) into subclasses on the basis of the lipoprotein particle size, indicated that any of the subclasses contained a higher ratio of TG to cholesterol in the subjects with higher HDL–LOOH compared with those with lower HDL–LOOH (Figure 3). Because TG-enriched particles have been reported to have a severely altered structure due to a “herniation” of the core TG to the HDL surface [5,43], they may be vulnerable to oxidative modification. However, a causal relationship between HDL–TG and oxidized HDL, which are two of the biomarkers for the assessment of functional HDL, has not yet been established.

## 4. Lipid Panel

Lipid markers are well-established predictors of vascular disease. The most frequently measured lipid markers are total cholesterol, HDL-C, LDL-C and TG (Table 1). In addition, non–HDL-C has been recommended as a marker to reflect the risk of vascular disease, because non–HDL-C is known to be more strongly correlated with cardiovascular disease than LDL-C [44,45]. The rationale for this recommendation is that non–HDL-C includes all cholesterol present in lipoprotein particles considered to be atherogenic, including LDL, lipoprotein(a), intermediate-density lipoprotein, and VLDL remnants [45]. Since ox-LDL is known to be involved in various diseases including cardiovascular diseases, and its concentrations in the circulating plasma and gingival crevicular fluid can be measured by using monoclonal antibodies, ox-LDL or the ratio of oxLDL to LDL is a useful biomarker for vascular disease [46].

Both high TG and low HDL-C levels impart a risk of heart disease. Previous studies have revealed that the ratio of TG to HDL-C is one of the major risk factors of cardiovascular diseases and insulin resistance [34,47,48]. Therefore, this TG/HDL-C ratio can be included in the lipid panel. In addition, properties of lipoproteins such as their particle size, number, and protein constituents, e.g., apoB and apoA-I, have attracted a great amount of attention as lipoprotein-related markers. For example, apoB shows a stronger association with the risk of coronary heart disease than non–HDL-C and LDL-C [44]. Thus, apoB is a useful marker to reflect the risk conferred by proatherogenic TG-rich VLDL, but its measurement is time-consuming and laborious compared with the measurement of non-HDL-C.

Many epidemiological studies have shown that high plasma concentrations of HDL-C are associated with a lower cardiovascular risk [3,4]. However, some studies have shown that HDL functionality is a better determinant of HDL-mediated cardiovascular protection than HDL-C concentration [8]. Thus, simple lipid tests to assess HDL functionality are clinically useful. In this study, we propose a new lipid profile, which includes two markers associated with HDL functionality, i.e., HDL–LOOH and HDL–TG (Table 1). This profile is expected to serve as an initial screening procedure for predicting approximate risks of cardiovascular disease.

Oxidized HDL has a pivotal role in the onset and progression of atherosclerotic plaque and cardiovascular disease, and HDL–LOOH measurement is a powerful way to assess oxidized HDL levels. ROS cause the peroxidation of HDL-lipids, and once lipid peroxidation is initiated, the propagation of chain reactions take place, thus resulting in an increase in HDL–LOOH. Several reactive intermediates (e.g., MDA) are formed as secondary products during lipid peroxidation, and they, together with LOOH, lead to the oxidative modification of HDL protein constituents such as apoA-I. Therefore, HDL–LOOH is a key molecule reflecting the oxidative status of HDL, and its measurement is useful for the assessment of oxidized HDL levels in the clinical laboratory. However, the HDL–LOOH /HDL-C ratio may be superior to HDL–LOOH content in predicting or indirectly assessing HDL functionality, because HDL function depends on how many dysfunctional HDL particles are present among the total HDL particles. 

Patients with carotid plaques show higher HDL–TG concentrations than those without them [5]. Furthermore, in contrast to HDL-C, HDL–TG is directly associated with metabolism and arteriosclerotic vascular alterations. Consequently, the HDL–TG content is a good marker for the assessment of HDL functionality and is recommended as a biomarker of metabolic and cardiovascular risk (Table 1). Because the TG/CE ratio in HDL particles is a critical factor for the structure and function of HDL, the HDL–TG/HDL-C ratio, instead of HDL–TG content, may be a more useful criterion for new diagnostic approaches in the assessment of lipoprotein-related development of atherosclerotic cardiovascular disease.

## 5. Conclusions 

Plasma HDL-C concentrations are not an appropriate marker of vascular effects of HDL and therefore do not represent a reliable therapeutic target. Recent studies have focused on measuring other indices of HDL, such as the functionality, size, or number of HDL-particle. Of all the indices, two markers associated with HDL functionality, i.e., HDL–LOOH and HDL–TG, are clinically available markers. A lipid panel including these two markers is expected to serve as an initial screening procedure for predicting approximate risks of cardiovascular disease. 

## Figures and Tables

**Table 1:** A new lipid panel for the assessment of lipoprotein-related atherosclerotic cardiovascular disease.

Recommended Lipid Panel
• Total cholesterol
• HDL cholesterol
• Triglyceride
• LDL cholesterol
• Non-HDL cholesterol (calculated)
• HDL-LOOH (HDL-LOOH /HDL-cholesterol)
• HDL-Triglyceride (HDL-Triglyceride/HDL-cholesterol)
Other markers 🞄 Total triglyceride/HDL-cholesterol 🞄 Total cholesterol/HDL-cholesterol 🞄 Lipoprotein particles (size, number) 🞄 Apoproteins (ApoB, ApoA-I) 🞄 Oxidized lipoproteins (ox-LDL, oxidized ApoA-I)
